# Impact of an IV proton pump inhibitor restriction policy on prescribing patterns and estimated drug cost

**DOI:** 10.3389/fphar.2026.1934624

**Published:** 2026-09-09

**Authors:** Ahmed R. N. Ibrahim, Saeed A. Alqahtani, Wael A. Alghamdi

**Affiliations:** 1 Department of Clinical Pharmacy, College of Pharmacy, King Khalid University, Abha, Saudi Arabia; 2 Faculty of Pharmacy, Minia University, Minia, Egypt; 3 Department of Pharmacy, Khamis Mushait General Hospital, Khamis Mushait, Saudi Arabia; 4 Aseer Health Cluster, Abha, Saudi Arabia

**Keywords:** drug cost, hospital pharmacy, prescribing patterns, proton pump inhibitors, restriction policy

## Abstract

**Introduction:**

Intravenous (IV) proton pump inhibitors (PPIs) are commonly used in hospitalized patients, although a substantial proportion of this use may be inappropriate, particularly when enteral therapy is feasible. Restriction policies may help optimize prescribing and reduce unnecessary drug costs. This study evaluated the impact of an IV PPI restriction policy on prescribing patterns and estimated direct drug cost at a regional secondary care hospital in Saudi Arabia.

**Methods:**

This retrospective before-and-after study was conducted from April 2023 to April 2024. The pre-restriction period was April to September 2023, and the post-restriction period was November 2023 to April 2024; October 2023 was excluded as the implementation washout month. Monthly hospital pharmacy records were used to collect IV and oral PPI prescriptions and recorded units, and prescribing and cost outcomes were normalized per 100 inpatient admissions. Clinical outcomes were not assessed. Pre- and post-restriction monthly values were compared using the Mann–Whitney U test.

**Results:**

After policy implementation, mean monthly IV PPI prescriptions per 100 inpatient admissions decreased from 92.1 to 37.8, a 59.0% reduction (p = 0.0131), whereas oral PPI prescriptions increased from 15.5 to 41.6 per 100 inpatient admissions, a 169.2% increase (p = 0.0202). Total PPI prescriptions decreased from 107.6 to 79.3 per 100 inpatient admissions, a 26.2% reduction (p = 0.0082). The proportion of IV PPI prescriptions declined from 85.6% to 45.0% (p = 0.0152). Mean estimated total PPI drug cost per 100 inpatient admissions decreased from 3,534.6 to 1,642.9 SAR, a 53.5% reduction (p = 0.0131).

**Conclusion:**

Implementation of an IV PPI restriction policy was associated with lower IV PPI prescribing, increased oral PPI use, and lower estimated direct drug cost. Clinical safety and efficacy were not evaluated and require further investigation.

## 1 Introduction

Proton pump inhibitors (PPIs) are among the most widely prescribed drug classes worldwide, with utilization increasing steadily across healthcare systems over the past 2 decades (Shanika et al., 2023). In hospitalized patients, PPIs are commonly used for acid-related disorders and for stress ulcer prophylaxis (SUP) in selected critically ill patients (Cook et al., 2024; MacLaren et al., 2025). However, substantial evidence indicates that PPIs, particularly intravenous (IV) PPIs, are often prescribed without an evidence-based indication or continued beyond the period in which acid-suppressive therapy is justified (Mohzari et al., 2020; Heidelbaugh et al., 2012; Targownik et al., 2022). Inappropriate IV PPI use has been documented in both ICU and non-ICU settings and represents an important and potentially modifiable source of avoidable inpatient drug expenditure (Alsultan et al., 2010; Craig et al., 2010; Nasser et al., 2010).

The clinical justification for IV PPI use is narrower than prescribing patterns often suggest. Oral PPIs have high bioavailability and provide effective acid suppression in patients with intact gastrointestinal function, while current guidance reserves IV administration for patients who cannot tolerate enteral therapy or who have a specific indication, such as active upper gastrointestinal bleeding (MacLaren et al., 2025; Targownik et al., 2022; Tringali et al., 2017; Amer et al., 2026). Despite this, IV formulations, which are often substantially more expensive than their oral equivalents, continue to account for a large proportion of inpatient PPI use in many settings (Mohzari et al., 2020; Ladd et al., 2014).

Inappropriate IV PPI prescribing has also been documented in Saudi Arabian hospitals in both ICU and non-ICU settings (Mohzari et al., 2020; Alsultan et al., 2010), yet local evidence on the effectiveness of institutional restriction policies remains limited. International studies suggest that targeted interventions, including formulary restriction, computerized prescriber order entry (CPOE) gating, audit-and-feedback, and pharmacist-led IV-to-oral conversion programs, can meaningfully influence prescribing behavior and reduce avoidable cost (Colombet et al., 2009; Bao et al., 2023; Lau et al., 2011; Wang et al., 2026; Patel et al., 2018). However, most published evaluations come from settings with different pricing structures, formulary controls, and prescribing environments. Whether a CPOE-based specialty restriction can achieve similar effects in a Saudi regional secondary care hospital has not been well studied. This study evaluated the impact of an IV PPI restriction policy implemented through the institutional electronic prescribing system on IV and oral PPI prescribing patterns and estimated direct PPI drug cost.

## 2 Methods

### 2.1 Study design and setting

This retrospective before-and-after study was conducted at a regional secondary care hospital in Saudi Arabia to evaluate the impact of an IV PPI restriction policy on prescribing patterns and estimated direct drug cost. The policy was implemented in October 2023. Data were collected over a 13-month period from April 2023 to April 2024. For the before-and-after comparison, the pre-restriction period was defined as April 2023 to September 2023 and the post-restriction period as November 2023 to April 2024. October 2023 was excluded because it represented the implementation month and was treated as a washout period to minimize contamination between the pre- and post-restriction periods. The study was approved by the Asir Institutional Review Board (approval number F10-2-2025).

### 2.2 Restriction policy

In October 2023, a CPOE-based restriction policy was initiated to reduce unnecessary IV PPI use and encourage oral PPI prescribing when the enteral route was feasible. During the initial implementation period, authorization to prescribe IV omeprazole remained available to Internal Medicine consultants and to several subspecialty consultants who were covering Internal Medicine services because gastroenterology coverage was not sufficient to assume full responsibility for IV PPI authorization. From January 2024, after adequate gastroenterology coverage became available, prescribing authorization was further restricted: gastroenterology consultants assumed responsibility for prescribing IV omeprazole following consultation when IV therapy was considered necessary. ICU and Emergency Medicine consultants remained exempt from this restriction and retained direct prescribing authorization. Other prescribers could not directly order IV omeprazole through the electronic prescribing system. No concurrent educational campaign, audit-and-feedback intervention, or formulary change was introduced during the study period. In addition, no major changes occurred in hospital bed capacity or institutional clinical guidelines that would be expected to independently influence PPI prescribing.

### 2.3 Data collection and outcome measures

Monthly hospital pharmacy records were used to collect the number of pharmacy-accepted PPI prescriptions and the corresponding recorded units for IV and oral formulations. Prescription counts did not include attempted orders blocked at the CPOE stage. Although the records captured medication quantities, actual administration to patients could not be confirmed; therefore, drug cost was reported as estimated direct drug cost. Hospital unit prices were obtained from the institutional medication price list. Prescribing outcomes included monthly IV, oral, and total PPI prescriptions, expressed per 100 inpatient admissions to account for variation in hospital activity, and the monthly proportion of IV PPI prescriptions out of total PPI prescriptions. Patient-level clinical data were not available; therefore, differences in patient case mix, prescribing indications, and clinical outcomes could not be assessed.

Economic outcomes included the monthly estimated direct drug cost of IV PPIs, monthly estimated direct drug cost of oral PPIs, and monthly total estimated direct drug cost of all PPIs. Estimated direct drug cost was calculated by multiplying the monthly number of PPI units recorded in hospital pharmacy records by the corresponding hospital unit price. Costs were expressed in Saudi Riyals (SAR). Accordingly, the economic analysis represents a partial direct-cost estimate based on medication utilization and does not include administration supplies, nursing time, pharmacy preparation, consultation workload, overhead, or downstream healthcare utilization. Estimated drug costs were also expressed per 100 inpatient admissions.

### 2.4 Outcomes and statistical analysis

The primary outcome was the monthly number of IV PPI prescriptions per 100 inpatient admissions before and after implementation of the restriction policy. Secondary outcomes included oral and total PPI prescriptions per 100 inpatient admissions, the proportion of IV PPI prescriptions out of total PPI prescriptions, and estimated direct drug cost per 100 inpatient admissions.

Monthly data were summarized using mean with standard deviation (SD) and median with interquartile range (IQR). Pre-restriction and post-restriction monthly values were compared using the Mann–Whitney U test. Given the limited number of monthly observations (six per period), the pre–post comparison was retained as the primary analysis; however, this comparison does not account for potential temporal dependence between consecutive monthly observations. To explore the potential influence of underlying temporal trends, exploratory segmented regression was additionally performed for monthly prescribing rates and estimated total PPI drug cost per 100 inpatient admissions, including terms for time, initiation of the restriction policy (level change), and time after implementation (change in trend). October 2023 was excluded from both analyses. Residual autocorrelation for the segmented regression of the primary outcome was assessed using the autocorrelation function and Ljung–Box test. Because of the short time series, the segmented analysis was considered exploratory and was not used as the primary basis for causal inference. Statistical analyses were performed using JMP® version 18 (SAS Institute, Cary, NC, United States). A two-sided p value of <0.05 was considered statistically significant.

## 3 Results

### 3.1 Prescribing patterns

Implementation of the IV PPI restriction policy was associated with marked differences in prescribing patterns (Table 1). Mean monthly IV PPI prescriptions decreased from 92.1 ± 14.2 to 37.8 ± 26.9 per 100 inpatient admissions (median [IQR], 88.7 [25.9] vs. 28.7 [46.4]), representing a 59.0% reduction (p = 0.0131). In contrast, mean monthly oral PPI prescriptions increased from 15.5 ± 5.7 to 41.6 ± 17.8 per 100 inpatient admissions (14.3 [10.3] vs. 44.8 [31.9]), a 169.2% increase (p = 0.0202). Mean monthly total PPI prescriptions also decreased from 107.6 ± 14.0 to 79.3 ± 10.0 per 100 inpatient admissions (106.0 [24.2] vs. 77.3 [17.3]), representing a 26.2% reduction (p = 0.0082).

**TABLE 1:** PPI prescribing and estimated direct drug cost before and after implementation of the IV PPI restriction policy.

Measure	Pre-restriction period		Post-restriction period		Absolute change	Relative change (%)	P-value
	Mean ± SD	Median (IQR)	Mean ± SD	Median (IQR)			
Prescriptions per 100 admissions							
IV PPI	92.1 ± 14.2	88.7 (25.9)	37.8 ± 26.9	28.7 (46.4)	−54.4	−59.0	0.0131
Oral PPI	15.5 ± 5.7	14.3 (10.3)	41.6 ± 17.8	44.8 (31.9)	+26.1	+169.2	0.0202
Total PPI	107.6 ± 14.0	106.0 (24.2)	79.3 ± 10.0	77.3 (17.3)	−28.2	−26.2	0.0082
IV PPI, % of total prescriptions	85.6 ± 5.2	87.3 (10.4)	45.0 ± 26.6	37.2 (46.5)	−40.6	—	0.0152
Estimated direct drug cost per 100 admissions (SAR)							
IV PPI	3,494.7 ± 654.3	3,448.3 (1,377.3)	1,518.5 ± 927.6	1,212.8 (1,150.8)	−1,976.2	−56.5	0.0131
Oral PPI	39.8 ± 7.6	41.3 (14.8)	124.4 ± 39.1	132.3 (53.8)	+84.6	+212.2	0.0051
Total PPI	3,534.6 ± 651.9	3,488.7 (1,378.6)	1,642.9 ± 892.4	1,350.2 (1,101.7)	−1,891.7	−53.5	0.0131

Absolute change was calculated as the post-restriction mean minus the pre-restriction mean. Relative change was calculated as [(post-restriction mean − pre-restriction mean)/pre-restriction mean] × 100.

PPI, proton pump inhibitor; IV, intravenous; SAR, saudi riyal; SD, standard deviation; IQR, interquartile range.

**FIGURE 1:**
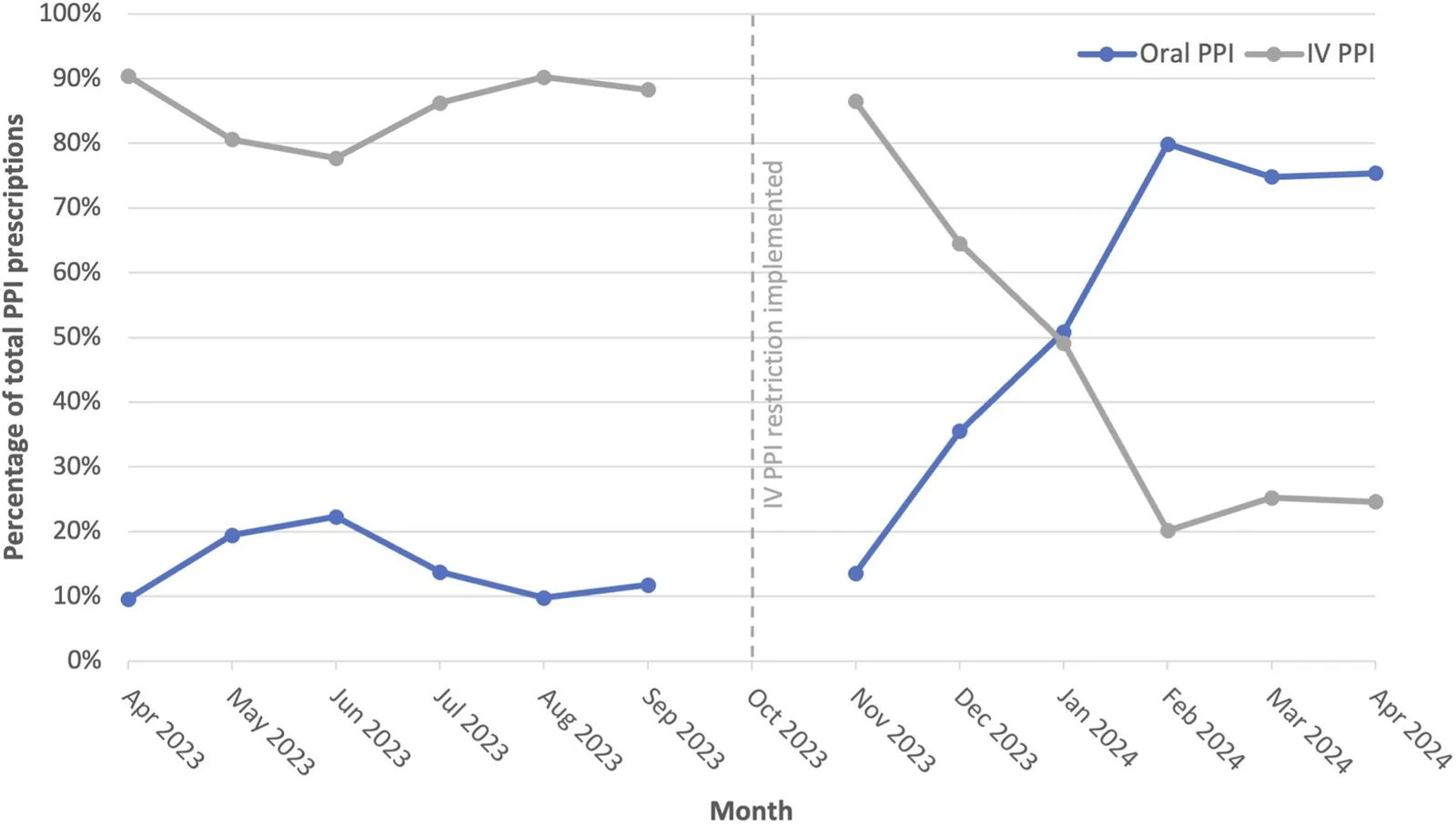
Monthly percentage of oral and IV PPI prescriptions before and after implementation of the IV PPI restriction policy. Monthly percentage of oral and IV PPI prescriptions from April 2023 to April 2024. October 2023 was the policy implementation month, was treated as a washout period, and was excluded from the pre- and post-restriction analyses.

The mean monthly proportion of IV PPI prescriptions declined from 85.6% before policy implementation to 45.0% after implementation, an absolute decrease of 40.6 percentage points (p = 0.0152). As shown in Figure 1, IV PPIs accounted for the predominant share of prescriptions throughout the pre-restriction period, whereas a progressive shift toward oral PPI prescribing was observed during the post-restriction period. The crossover point occurred around January 2024, after which oral PPI prescriptions exceeded IV PPI prescriptions and remained predominant throughout the remainder of follow-up. In the exploratory segmented regression of IV PPI prescriptions per 100 inpatient admissions, there was no significant baseline trend (−3.75 per 100 inpatient admissions inpatient per month; p = 0.261) or immediate level change following initiation of the restriction (+1.00; p = 0.950). The post-intervention trend decreased by an additional 9.39 prescriptions per 100 inpatient admissions per month relative to the baseline trend, although this did not reach statistical significance (p = 0.065). No evidence of first-order residual autocorrelation was observed (lag-1 autocorrelation, −0.044; Ljung–Box p = 0.865).

### 3.2 Estimated direct drug cost

Mean monthly estimated IV PPI drug cost decreased from 3,494.7 ± 654.3 to 1,518.5 ± 927.6 SAR per 100 inpatient admissions, representing a 56.5% reduction (p = 0.0131) (Table 1). In contrast, mean monthly estimated oral PPI drug cost increased from 39.8 ± 7.6 to 124.4 ± 39.1 SAR per 100 inpatient admissions, a 212.2% increase (p = 0.0051). Overall, mean monthly estimated total PPI drug cost decreased from 3,534.6 ± 651.9 to 1,642.9 ± 892.4 SAR per 100 inpatient admissions, representing a 53.5% reduction (p = 0.0131).

In exploratory segmented regression of total estimated PPI drug cost per 100 inpatient admissions, the pre-intervention trend was downward but not statistically significant (−147.8 SAR per 100 inpatient admissions per month; p = 0.331). No significant immediate level change (−104.8 SAR per 100 inpatient admissions; p = 0.885) or change in post-intervention trend (−257.1 SAR per 100 inpatient admissions per month; p = 0.239) was detected.

**FIGURE 2:**
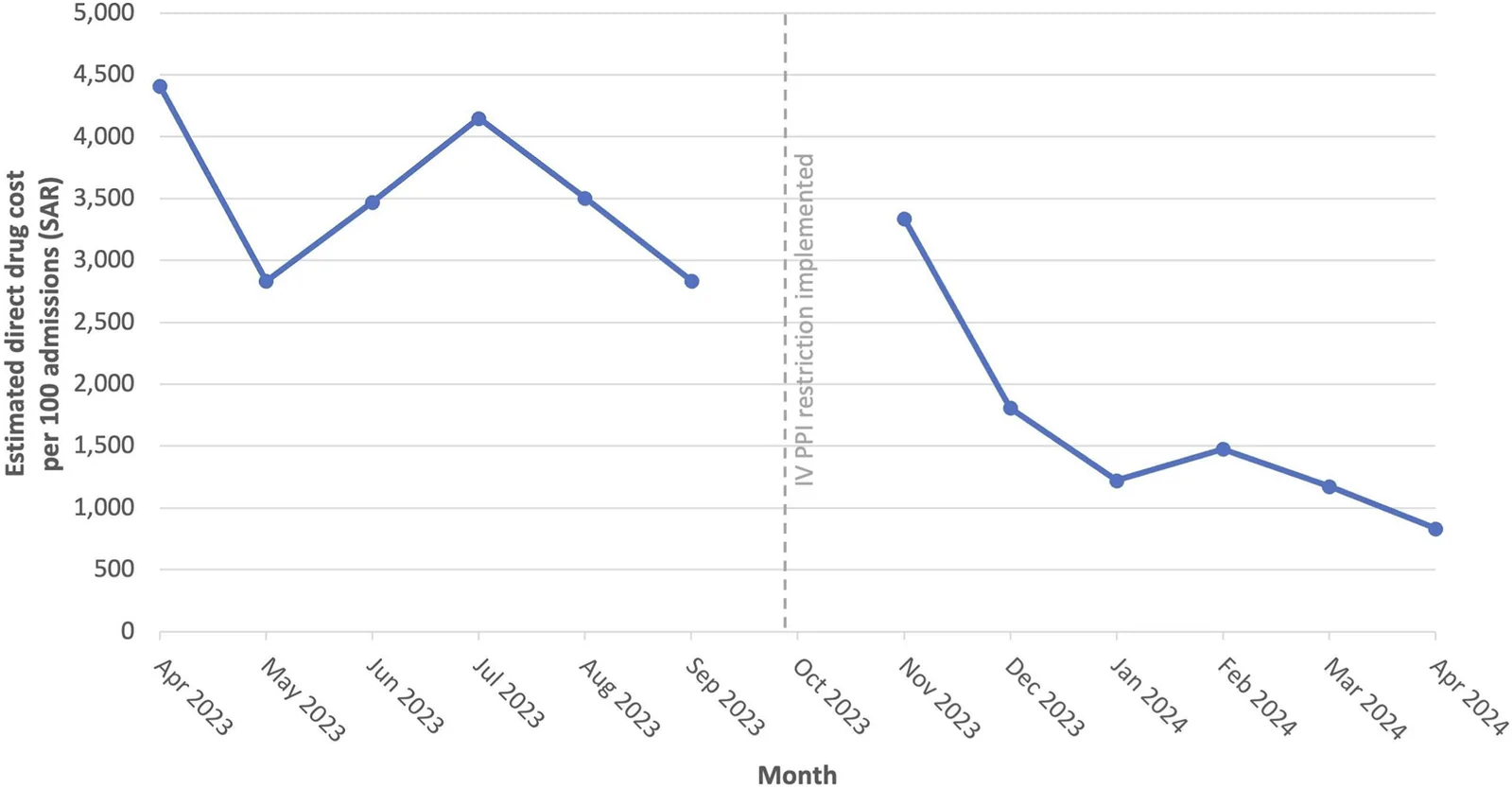
Monthly estimated direct PPI drug cost per 100 inpatient admissions before and after implementation of the IV PPI restriction policy. Monthly estimated direct PPI drug cost per 100 inpatient admissions from April 2023 to April 2024. Cost was estimated using hospital unit prices and the number of PPI units recorded in hospital pharmacy records. October 2023 was the policy implementation month, was treated as a washout period, and was excluded from the pre- and post-restriction analyses.

As shown in Figure 2, total estimated PPI drug cost fluctuated during the pre-restriction period and generally declined across the post-restriction period.

## 4 Discussion

In this before-and-after evaluation of a CPOE-based specialty restriction policy at a Saudi regional secondary care hospital, implementation of the policy was associated with substantial differences in PPI prescribing patterns and estimated direct drug cost. IV PPI prescribing decreased by 59.0% per 100 inpatient admissions, while the proportion of IV PPI prescriptions declined from 85.6% to 45.0%. In parallel, oral PPI prescribing increased, indicating a marked shift toward the oral route. Total PPI prescribing per 100 inpatient admissions was 26.2% lower during the post-restriction period, while estimated total PPI drug cost per 100 inpatient admissions was 53.5% lower.

The magnitude of the reduction in IV PPI prescribing was substantial in comparison with changes reported in prior stewardship interventions. Colombet *et al.* reported a sustained but more modest shift in the IV-to-oral PPI ratio after a multifaceted oral-route promotion program in a French university hospital (Colombet et al., 2009). Bao *et al.* found that a pharmacist-led IV-to-oral conversion program in a Chinese cardiovascular surgery population reduced inappropriate IV PPI use (Bao et al., 2023). More recently, Wang *et al.* showed that policy-based PPI stewardship interventions generally produced larger and more durable effects than education (Wang et al., 2026). Unlike some previously reported interventions, the present policy used a CPOE-based restriction with progressively narrower prescribing authorization, culminating in predominantly gastroenterology-directed IV PPI prescribing from January 2024, while ICU and Emergency Medicine consultants retained direct prescribing access.

The reduction in total PPI prescribing should be interpreted cautiously. Although total PPI use per 100 inpatient admissions was 26.2% lower during the post-restriction period, patient-level indication data were unavailable to determine whether this reflected changes in prescribing appropriateness or other factors. Moreover, the exploratory segmented analysis indicated that total PPI utilization was already declining before policy implementation, without a detectable additional change in trend afterward. Therefore, the observed reduction in total PPI prescribing cannot be specifically attributed to the restriction policy.

These findings are consistent with current guidance, which limits IV PPI use to selected patients and emphasizes IV-to-oral conversion whenever feasible (MacLaren et al., 2025; Targownik et al., 2022; Amer et al., 2026). The observed shift from IV toward oral PPI prescribing is directionally consistent with guidance emphasizing oral therapy when the enteral route is feasible.

From an economic perspective, mean estimated total PPI drug cost per 100 inpatient admissions was 53.5% lower during the post-restriction period. However, the exploratory segmented analysis did not identify a statistically significant immediate level or trend change in total estimated drug cost following initiation of the restriction policy. Accordingly, the observed pre–post cost difference cannot be attributed solely to the restriction policy. The economic analysis was limited to utilization-based drug cost and does not account for administration-related costs or the potential administrative and consultation burden associated with gastroenterology authorization. Therefore, the findings should not be interpreted as a comprehensive estimate of the policy’s overall economic impact. Consistent with reports from Saudi Arabia (Mohzari et al., 2020), Lebanon (Nasser et al., 2010), and the United States (Ladd et al., 2014; Ahrens et al., 2012), our findings indicate that reductions in IV PPI utilization may be accompanied by substantial reductions in direct drug cost in settings where IV PPIs are more costly than their oral equivalents.

This study has several limitations. First, the before-and-after design and short observation period limit causal attribution. Although prescribing outcomes were normalized to inpatient admissions and exploratory segmented regression was performed to examine underlying temporal trends, only six monthly observations were available before and after implementation. In addition, prescribing authorization was further narrowed in January 2024, meaning that the intervention was not static throughout the post-restriction period. This limited statistical power and precluded robust assessment of seasonality and more complex autocorrelation structures. Accordingly, the observed pre–post differences should be interpreted as associations with policy implementation rather than definitive causal effects. Longer time-series analyses, ideally incorporating a concurrent control site, would provide stronger evidence of intervention effectiveness. Second, the analysis relied on aggregated monthly pharmacy data rather than patient-level prescribing records. Although normalization per 100 inpatient admissions accounted for variation in hospital activity, the data did not permit adjustment for patient acuity, case mix, prescribing indications, or treatment duration. Third, the cost analysis was limited to estimated direct drug cost and did not include administration-related costs, adverse-event costs, or downstream effects on length of stay. Fourth, patient-level clinical outcomes were not available; therefore, the safety and clinical consequences of the restriction policy could not be assessed. Further studies incorporating patient-level outcomes are needed to establish the clinical safety and effectiveness of this approach. Finally, this was a single-center study conducted at a regional secondary care hospital, which may limit generalizability to other hospital settings.

In summary, a CPOE-based specialty restriction on IV PPI prescribing was associated with lower IV PPI utilization, increased oral PPI prescribing, and lower estimated direct PPI drug cost per 100 inpatient admissions. These findings suggest that CPOE-based route restrictions may represent a useful component of hospital acid-suppression stewardship; however, the short observation period and absence of patient-level clinical outcomes limit causal and clinical interpretation. Longer studies incorporating patient-level safety and clinical outcomes are needed to better establish the effectiveness and safety of this approach.

## Data Availability

The datasets presented in this article are not readily available due to institutional and ethical restrictions. Requests to access the datasets should be directed to aribrahim@kku.edu.sa.
